# Aggregation of PolyQ Proteins Is Increased upon Yeast Aging and Affected by Sir2 and Hsf1: Novel Quantitative Biochemical and Microscopic Assays

**DOI:** 10.1371/journal.pone.0044785

**Published:** 2012-09-06

**Authors:** Aviv Cohen, Liron Ross, Iftach Nachman, Shoshana Bar-Nun

**Affiliations:** Department of Biochemistry and Molecular Biology, George S. Wise Faculty of Life Sciences, Tel Aviv University, Tel Aviv, Israel; Brown University, United States of America

## Abstract

Aging-related neurodegenerative disorders, such as Parkinson's, Alzheimer's and Huntington's diseases, are characterized by accumulation of protein aggregates in distinct neuronal cells that eventually die. In Huntington's disease, the protein huntingtin forms aggregates, and the age of disease onset is inversely correlated to the length of the protein's poly-glutamine tract. Using quantitative assays to estimate microscopically and capture biochemically protein aggregates, here we study in *Saccharomyces cerevisiae* aging-related aggregation of GFP-tagged, huntingtin-derived proteins with different polyQ lengths. We find that the short 25Q protein never aggregates whereas the long 103Q version always aggregates. However, the mid-size 47Q protein is soluble in young logarithmically growing yeast but aggregates as the yeast cells enter the stationary phase and age, allowing us to plot an “aggregation timeline”. This aging-dependent aggregation was associated with increased cytotoxicity. We also show that two aging-related genes, *SIR2* and *HSF1*, affect aggregation of the polyQ proteins. In Δ*sir2* strain the aging-dependent aggregation of the 47Q protein is aggravated, while overexpression of the transcription factor Hsf1 attenuates aggregation. Thus, the mid-size 47Q protein and our quantitative aggregation assays provide valuable tools to unravel the roles of genes and environmental conditions that affect aging-related aggregation.

## Introduction

Huntington's disease (HD) is a progressive neurodegenerative disorder manifested by dysfunction and cell death mainly in the striatal and cortical brain regions [1]–[3]. HD patients experience deteriorating symptoms including chorea, progressive dementia, psychiatric manifestations, and death within 15 years of symptoms onset. Currently there is neither cure nor treatment to postpone the onset of HD or to slow its progression [4]–[6]. HD is an autosomal dominant genetic disease, caused by mutations in the first exon of the huntingtin gene, *Htt*, that increase the number of CAG repeats to generate expanded polyglutamine (polyQ) tracts in the N-terminal region of the huntingtin protein [7].

It is still unclear why the ubiquitously expressed huntingtin has a distinctive neurological phenotype and if huntingtin's loss-of-function leads to HD [8]; [9]. The widely accepted notion is that HD is caused by a harmful gain-of-function of expanded polyQ since mice models with mutated huntingtin exon 1 recapitulate HD symptoms, including neuronal dysfunction and death [10]. Hence, although CAG repeat RNA was recently proposed as an auxiliary toxic agent in polyQ disorders [11], it appears that the deleterious effects in HD are mostly related to the tendency of the mutated huntingtin protein to misfold and aggregate. This property is observed with purified proteins and in neurons, where cellular aggregates are mainly composed of huntingtin [12]. *In vivo* and *in vitro* studies of polyQ fibrillogenesis indicate that aggregation is an intrinsic feature of long polyQ tracts and this tendency increases with the length of the polyQ repeat [13]; [14]. Indeed, HD is one of nine known polyQ repeat autosomal dominant disorders, all of which result from mutations that expand the polyQ tract within different but specific proteins [15]–[18]. Although the mutated proteins have no shared qualities beside the polyQ mutation, all these disorders are characterized by progressive neurodegeneration as well as the formation of polyQ protein aggregates.

The common risk factor of all aggregation diseases is aging [19]–[21] and, in HD, the combination of aging and genetic mutations is manifested by inverse correlation between the age of disease onset and the number of CAG repeats. Repeats shorter than 35Q are asymptomatic, whereas repeats exceeding the 40Q threshold ensure HD development such that 40–50Q give rise to the normal adult-onset of HD while longer repeats result in juvenile cases [22]–[27]. Although various statistical models strongly correlate the mean age of HD onset with the length of the CAG repeat [28]; [29], patients with the same polyQ length exhibit very different ages of onset. Hence, modifiers of HD and aggregation, apart from the length of the polyQ, must be implicated [5]. Based on screening HD patients or model organisms, combinations of genetic and environmental factors clearly affect the age of HD onset [22].

Cellular aging is considered as a progressive decline in the proteostasis machinery and the response to changing environment. Aging is caused partly by genetic factors accompanied by metabolic, environmental and stochastic factors [30]; [31]. Progress in cellular aging research is driven by single-celled eukaryotes such as the budding yeast *Saccharomyces cerevisiae*. In this organism, genetic modulators of replicative lifespan (RLS) are being identified, and chronological lifespan (CLS) is considered useful for understanding the aging process in non-dividing mammalian cells such as neurons [32]; [33]. Importantly, a subset of pathways that influence longevity in yeast is conserved in other eukaryotes, including mammals. A genome-wide screen in *S. cerevisiae* has identified CLS-affecting genes that are highly conserved in other species, suggesting that longevity is a fundamental process conserved in evolution [34]. Finally, the stationary phase model of aging in yeast recapitulates many pathological alterations observed during neuronal aging, highlighting the power of yeast as a model system to explore the molecular basis of aging–related diseases of the central nervous system [35].

Among the conserved genes implicated in determining lifespan are the sirtuins, a family of class III NAD+-dependent protein deacetylases. In their unique lysine deacetylation reaction, NAD+ is cleaved, 29-O-acetyl-ADP-ribose is generated and nicotinamide (NAM) is released. Silent information regulator 2 (Sir2) in yeast or its closest mammalian homolog Sirt1 are conserved from yeast to mammals and are shown to regulate metabolism and longevity [36]–[38]. RLS in yeast is extended when Sir2 is overexpressed and decreases upon *SIR2* deletion [39]. Also, lifespan extension associated with dietary restriction (DR) requires Sir2 and NAD+ since it is annulled by deletion of either *SIR2* or *NPT1* (a gene in the NAD^+^ biosynthetic pathway). However, the role of sirtuins in DR-mediated lifespan extension is controversial; in yeast at stationary phase, Sir2 actually blocks extreme CLS extension mediated by DR [40].

Heat shock factor 1 (Hsf1), a master regulator of transcription, is another highly conserved protein that plays an important role in longevity as well as in maintaining proteostasis and adequate response to proteotoxic stresses. In *Caenorhabditis elegans*, Hsf1 is required for enhanced thermotolerance and suppression of proteotoxicity and was also shown to regulate aging [41]; [42] and to affect lifespan extension by DR [43]. Hsf1 is a regulator of lifespan also in yeast, as the CLS extender Ecl2 in *Schizosaccharomyces pombe* and its functional homolog YGR146C in *S. cerevisiae* are direct targets of Hsf1 and overexpression of Hsf1 in fission yeast extends CLS [44]. Hsf1 is activated by heat-shock, oxidative, metabolic or environmental stresses, although our understanding of Hsf1 regulation remains incomplete. In yeast, under normal growth conditions, Hsf1 is a constitutively phosphorylated homotrimer but inactive. Upon exposure to stress, Hsf1 is hyperphosphorylated and adopts an active conformation. Hsf1 binds to heat shock response elements (HSE) in the promoter region of its target genes and activates their expression [45]–[48]. Among the many Hsf1 target genes are the molecular chaperones, which resolve damaged/misfolded and aggregated proteins generated by heat stress or metabolism [49]; [50]. Interrelations between Hsf1 and Sirt1, the closest mammalian homolog of Sir2, may underlie their effects on proteostasis. Sirt1 phosphorylation is required for cell survival under stress, and mammalian Hsf1 is one of the substrates that phosphorylated Sirt1 deacetylates and co-activates, allowing the deacetylated Hsf1 to bind to HSE and activate the molecular chaperones network [51]–[53].

Among the genetically manipulatable model organisms for studying aggregation and toxicity of polyQ proteins, *S. cerevisiae* provides unsurpassed tools to decipher disease-associated cellular processes and identify novel therapeutic targets [54]. Although this unicellular eukaryote neither resembles neurons nor expresses endogenous huntingtin, the relevant cellular pathways appear to be highly conserved between humans and yeast. Hence, yeast is an established system for studying the causes and consequences of polyQ aggregation, and to address aging, DR and oxidative stress at the cellular and molecular levels [33]; [35]; [55]–[67]. Here we follow the effects of aging and altered expression or activity of *SIR2* and *HSF1* on the aggregation of polyQ proteins in *S. cerevisiae*. Our most interesting results are with the 47Q that harbors a mid-size polyQ tract characteristic of the threshold of HD onset. We find that 47Q is non-toxic and soluble in growing yeast, but aggregates as cells age. Upon *SIR2* deletion, the aging-dependent aggregation is aggravated, while overexpression of Hsf1 attenuates aggregation. Hence, Sir2 and Hsf1 appear to affect aggregation-related processes during aging.

## Results

### Quantitative measurements of polyQ proteins aggregation in yeast cells

**Figure 1:**
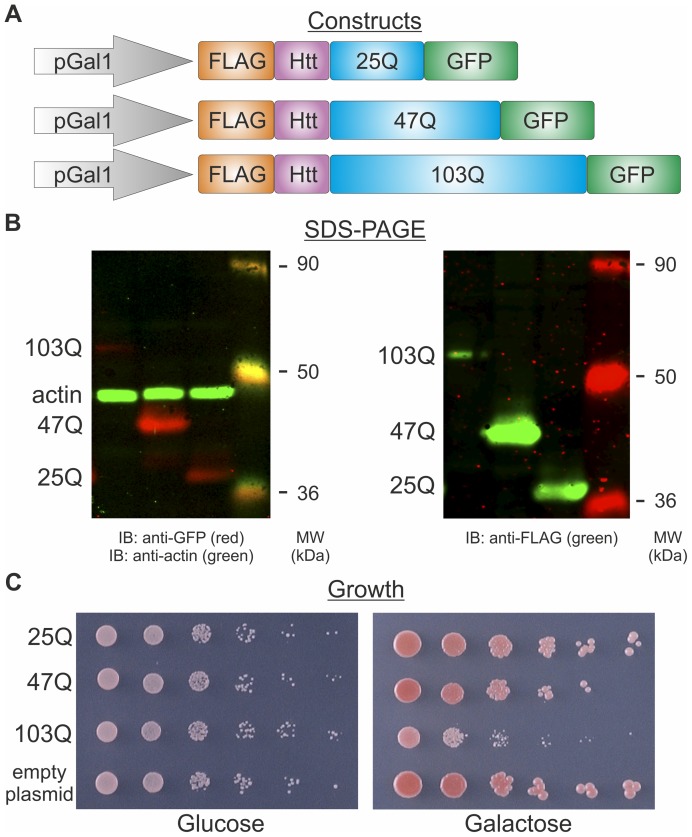
Proteins with polyQ of different lengths show different characteristics. A Schematic representation of the constructs used to express polyQ proteins. Each contains a FLAG tag at its N-terminus, followed by huntingtin's exon 1 encoding the first 17 amino acids (Htt), a polyQ tract of different length, and GFP at the C-terminus. Expression of all polyQ proteins is driven by the pGal1 promoter that is induced by galactose and repressed by glucose. B Wild-type cells (W303–1b) expressing the 25Q, 47Q or 103Q were grown logarithmically (0.6–1 A_600_) for 8 hours under galactose induction. Cell lysates were resolved by SDS-PAGE and polyQ proteins were detected by immunoblotting (IB) with a mouse anti-FLAG antibody followed by IRDye 800CW-conjugated goat anti-mouse IgG (right panel) or a rabbit anti-GFP followed by Dylight 680-labeled goat anti-rabbit IgG (left panel, red). A mouse anti-actin followed by IRDye 800CW-conjugated goat anti-mouse IgG was used as a loading control (left panel, green). Blots were visualized by the Odyssey Infrared Imaging System. C Wild-type cells (W303–1b) expressing the 25Q, 47Q, 103Q or an empty plasmid were grown logarithmically in glucose and 10-fold serial dilutions (starting with 7.5×10^6^ cells) were spotted on glucose or galactose plates.

Aggregates of polyQ proteins and their distribution in live cells are usually detected by fluorescence microscopy, owing to a fluorescent tag attached to the polyQ proteins [55]; [56]. Although aggregation patterns differ from cell to cell, images are usually evaluated qualitatively and any quantitative analysis merely determines how many cells in a population contain aggregates of polyQ proteins [56]. In this study we express in yeast, under galactose induction, GFP- and FLAG-tagged polyQ proteins harboring 25, 47 or 103 glutamine residues (referred to as 25Q, 47Q and 103Q), to demonstrate the basic characteristics conferred by the different polyQ lengths (Figure 1A, B). Among them, aggregation-prone polyQ proteins are toxic to different organisms, including yeast [57]. Indeed, upon galactose induction, expression of the 103Q inhibits cell growth, whereas 25Q expression does not (Figure 1C). A calculated duplication time of less than 2 hrs for cells harboring polyQ plasmids when grown in glucose was extended to around 4 hrs for cells expressing 25Q and to around 10 hrs for cells expressing 103Q when grown in galactose.

**Figure 2:**
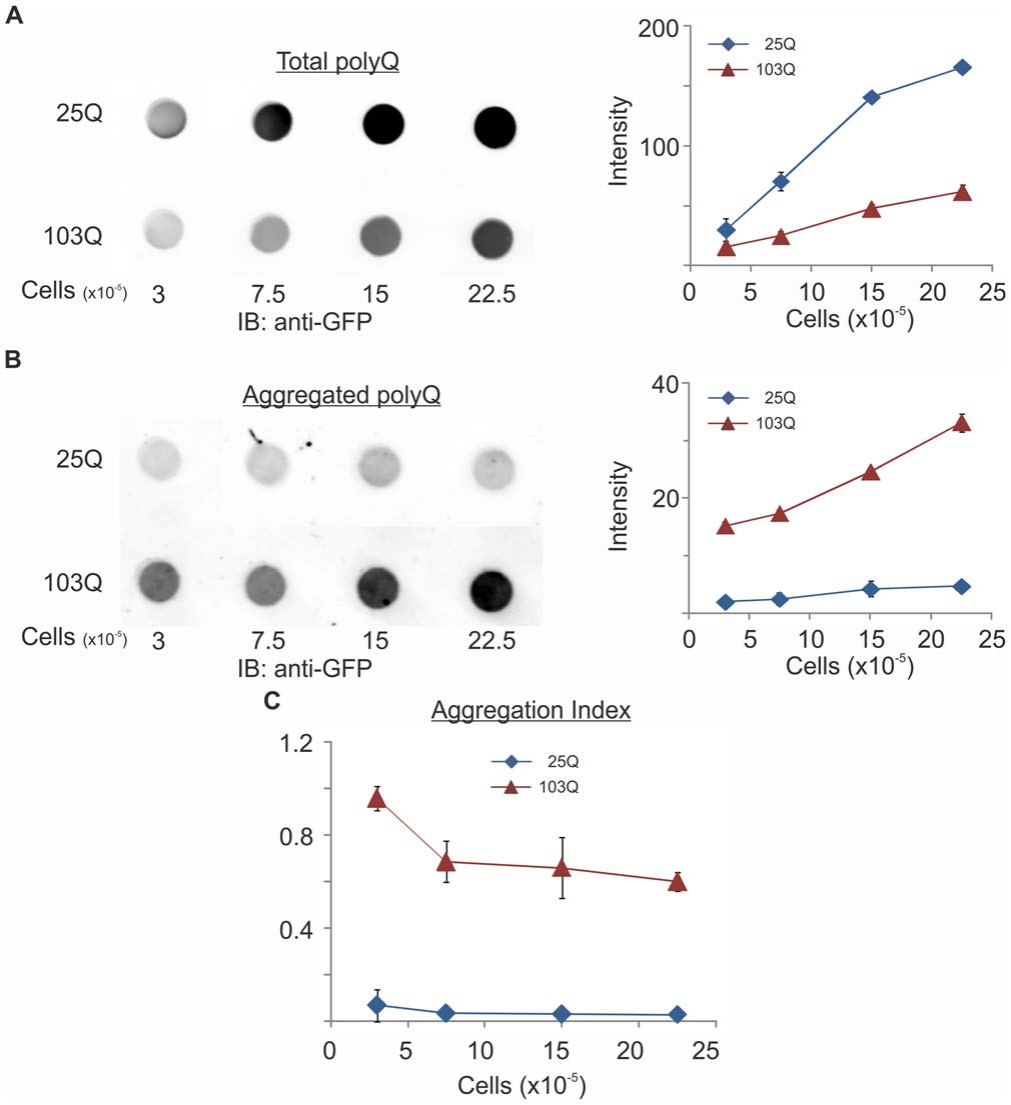
Quantitative filter retardation assay for polyQ proteins aggregates. Wild-type cells (KFY100) expressing 25Q or 103Q were grown for 3 days under galactose induction. Cells were lysed and lysates from the indicated number of cells were A adsorbed onto untreated nitrocellulose to estimate the total amount of polyQ proteins, or B SDS was added and lysates were filtered through SDS-soaked membranes to capture polyQ aggregates. On both membranes, polyQ proteins were quantified by immunoblotting (IB) with a rabbit anti-GFP antibody followed by Dylight 680-labeled goat anti-rabbit IgG and visualized (only one set of transformants is presented) and quantified by the Odyssey Infrared Imaging System. C Aggregation Index (in arbitrary units) was calculated as the ratio between the filtered (panel B) and absorbed (panel A) polyQ proteins. Data from 3 independent transformants are presented as mean ± SE.

Aggregates of polyQ proteins are formed in cells under different circumstances and are correlated with cytotoxicity. To gain insights into the various factors that affect aggregation, we developed a reproducible, quantitative and linear method to measure the amount of aggregates in yeast cells. This method takes advantage of the insolubility of polyQ aggregates in sodium dodecyl sulfate (SDS) [13]. Briefly, following cell lysis, samples are divided into two aliquots. One aliquot is adsorbed directly onto a nitrocellulose membrane to estimate total amounts of polyQ proteins, whereas SDS up to 2% (w/v) is added to the other aliquot that is filtered through a SDS-soaked membrane to capture the polyQ aggregates. While high concentration of SDS that denatures and charges proteins prevents their adsorption, aggregates are resistant to SDS and are retarded on the membrane. By immunoblotting we quantify the amounts of total polyQ proteins adsorbed to the untreated membrane (Figure 2A) and the aggregates retarded on the SDS-treated membrane (Figure 2B). These amounts are linear over a 10-fold range of cell lysates applied, thus allow calculating an “aggregation index” as the ratio of the aggregates to the total polyQ proteins (Figure 2C). Clearly, while both 25Q and 103Q are expressed (Figures 1B and 2A), aggregates are detected only for 103Q and not for 25Q (Figure 2B, C).

### Increased aggregation of long and mid-size polyQ protein is observed upon aging

**Figure 3:**
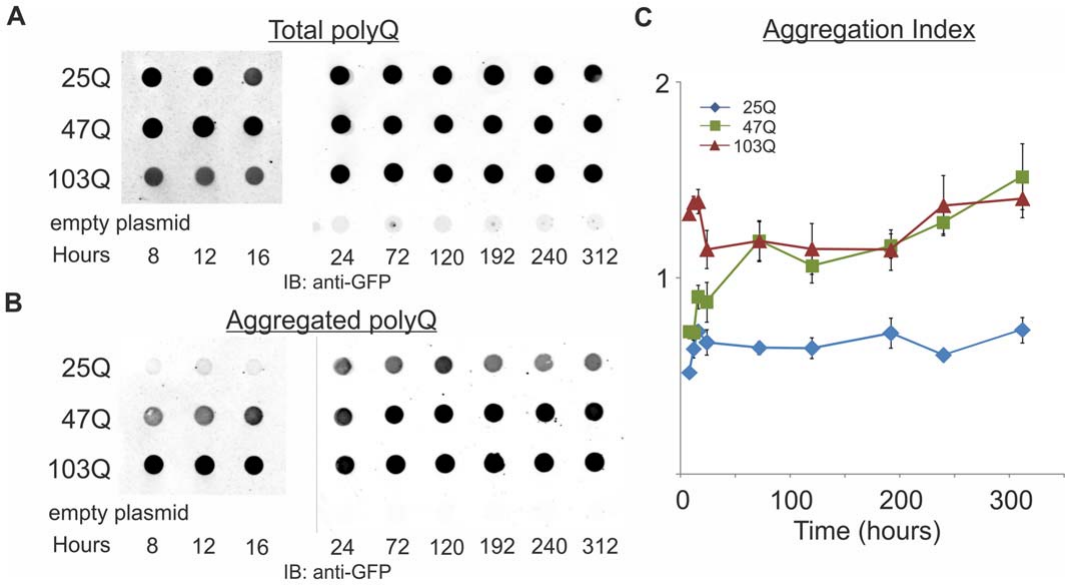
Quantitative retardation assay reveals that aggregation of 47Q increases upon aging. Wild-type cells (W303–1b) expressing 25Q, 47Q or 103Q were grown under galactose induction for the indicated time, and at each time point 11.25×10^5^ cells (within the linear range; see Figure 2) were lysed. Early time points (8–16 hrs) were done separately from later time points. Total amounts A and captured aggregates B of polyQ proteins were quantified and Aggregation Index C was calculated as described in and in Figure 2. Data from 3 independent transformants, 3 triplicates each, are presented as mean ± SE. Statistical significance between the different polyQ was calculated by split-plot ANOVA. We found p<0.0001 between 25Q and 47Q; p<0.001 between 47Q and 103Q; and no significance between experiments of each polyQ.

The quantitative analysis allows us to monitor the aggregation of the various polyQ proteins during cell aging and draw a timeline for the aggregation process. Aggregate formation of the long 103Q occurs very early and it somewhat increases as the cells age, while the short 25Q does not aggregate at any age (Figure 3B, C). Importantly, the mid-size 47Q does not aggregate in logarithmically growing young cells, but its aggregation is markedly increased as the cells age (Figure 3B, C). The 47Q aggregation starts when the cells enter the stationary phase and stop dividing and progresses with age (Figure 3C).

**Figure 4:**
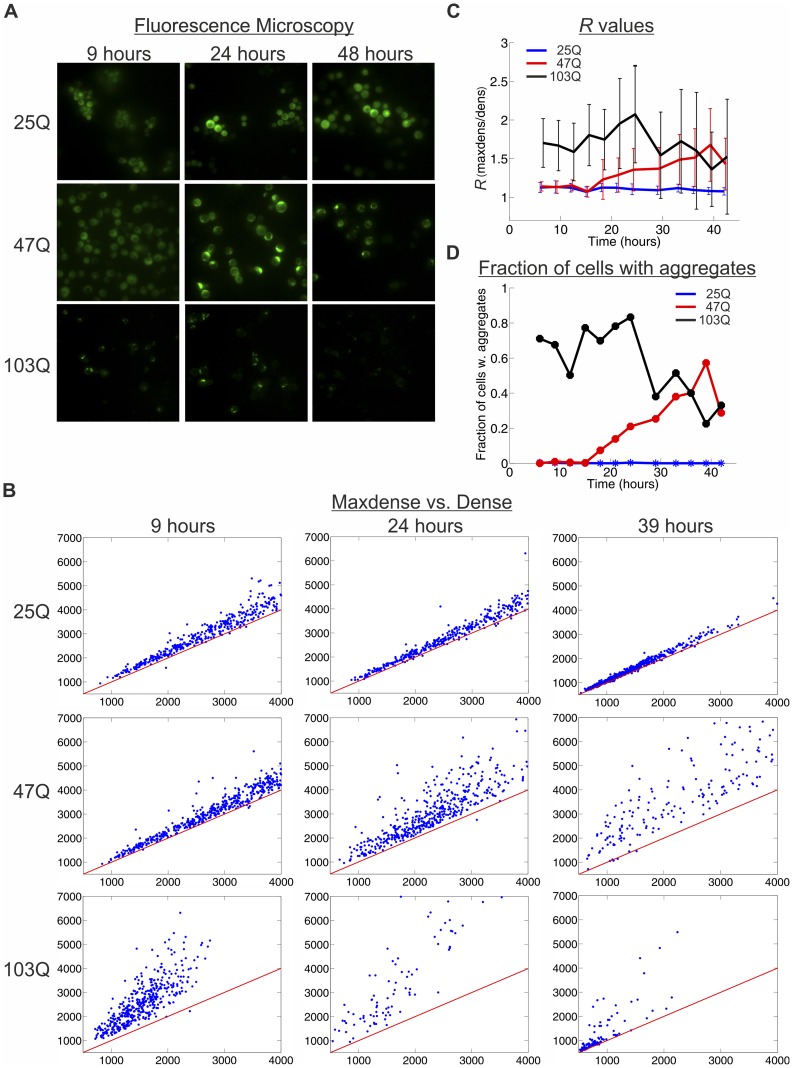
Quantitative microscopic assay reveals that aggregation of 47Q increases upon aging. A Wild-type cells (W303–1b) expressing 25Q, 47Q or 103Q were grown under galactose induction for up to 42 hrs. At each time point hundreds of cells were imaged by fluorescence microscopy. Images of 3 representative time points are presented. B Microscopic images from a similar experiment were analyzed for the presence of aggregates in individual cells as presented by the ratio between the maximal density (y axis) and the density (x axis) and 3 representative time points are presented (for all time points, see Figures S1, S1 and S3). C The ratio *R* between the maximal density and the density throughout the experiment plotted over time. At each time point the mean values ± SD are shown and the error bars reflect the variability between individual cells. D The fraction of cells with aggregates (defined as cells with *R* above a cutoff of 1.5) plotted over time.

This aggregation of the various polyQ proteins and especially the aging-dependent change in 47Q aggregation is strongly corroborated by our quantitative fluorescence microscopy. To quantify the aggregation level within individual cells, we analyze in hundreds of cells per sample the ratio *R* between the GFP fluorescence in the brightest focus and the average GFP fluorescence in the whole cell (Figure 4 A, B and Figures S1, S2 and S3). In 25Q-expressing cells with no aggregates this ratio is close to 1 (Figures 4B and S1), 103Q-expressing cells with aggregates show ratio values higher than 1 (Figures 4B and S3), and in 47Q-expressing cells the ratio increases upon aging (Figures 4B and S2). When *R* is plotted along the yeast aging process (Figure 4C), this ratio is in striking agreement with the Aggregation Index determined by filtration (Figure 3C). By defining cells with *R* above a cutoff of 1.5 as having aggregates, we calculated the fraction of cells with aggregates (Figure 4D). Both quantitative approaches reflect the aging-dependent aggregation of the 47Q, with no aggregation of the 25Q and immediate onset of aggregation of the 103Q. While this manuscript was under revision, a study in *C. elegans* reported the *in vivo* dynamics of polyQ aggregation using fluorescence microscopy, fluorescence recovery after photobleaching and fluorescence correlation spectroscopy [68]. Also in that study, the brightest foci represented aggregates, as they were immobile. The decline in *R* of 103Q at late time points stems from an age-dependant decline of total GFP fluorescence in these cells, as shown in Figures 4B and S3. Since this decline is not observed in the filter retardation assay (Figure 3), it is likely not the consequence of aggregates loss. Combined, our two quantitative analyses indicate that the mid-size 47Q is soluble in growing yeast and aggregates progressively as the yeast age.

### Upon *SIR2* deletion, the aging-dependent aggregation is aggravated, while overexpression of Hsf1 attenuates aggregation

**Figure 5:**
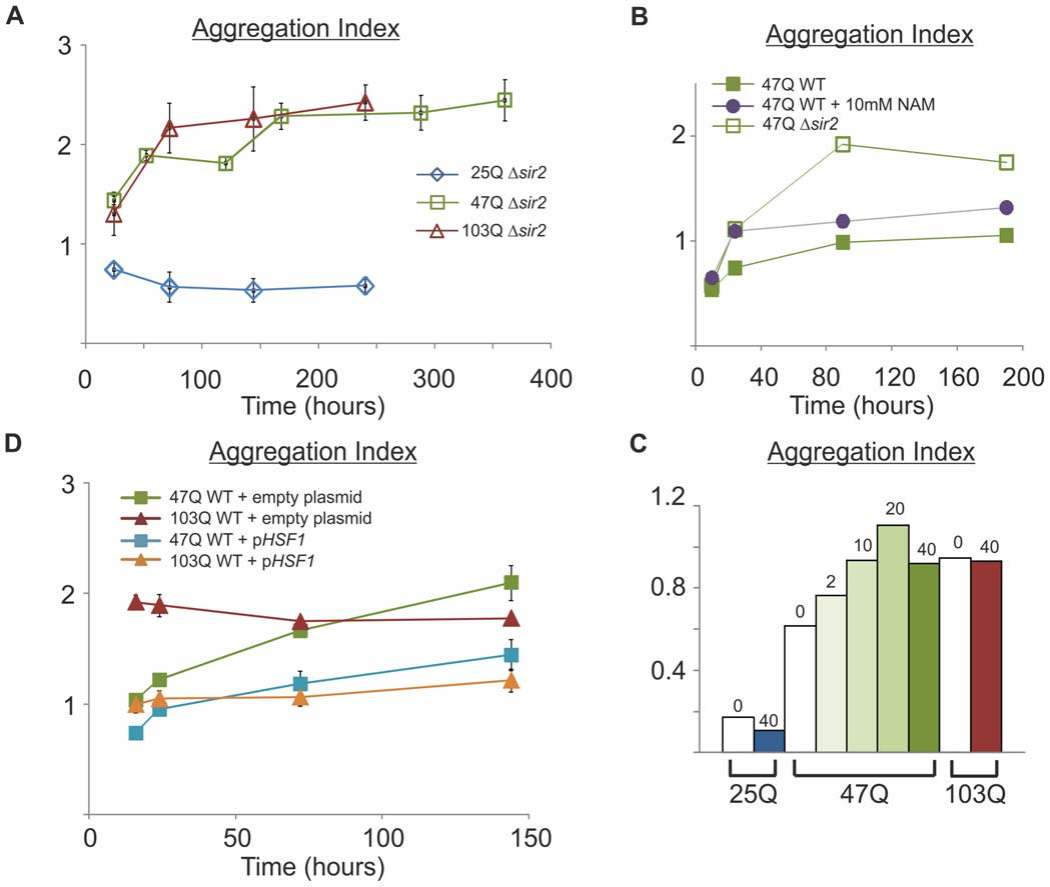
Sir2 and Hsf1 are involved in the aging-dependent aggregation of polyQ proteins. A Δ*sir2* (RS1717; W303–1b *sir2*Δ::*his5* ^+^) cells expressing 25Q, 47Q or 103Q were grown under galactose induction for the indicated time, collected, lysed, and Aggregation Index was calculated for each time point, as described in Figure 3. Data from 3 independent transformants, 3 triplicates each, are presented as mean ± SE. B Wild-type (WT, W303–1b) or Δ*sir2* cells expressing 47Q were grown for the indicated time under galactose induction. Wild-type cells were also exposed to 10mM nicotinamide (NAM). Cells were, collected and lysed and Aggregation Index for each time point was calculated as described in Figure 3. C Wild-type cells (W303–1b) expressing the indicated polyQ proteins were grown under galactose induction for 72 hours with the indicated concentration (mM) of NAM. Cells were collected, lysed and Aggregation Index was calculated as described in Figure 3. D Wild-type (W303–1b) cells expressing the indicated polyQ proteins and harboring either an empty (pRS314) or p*HSF1* plasmid were grown under galactose induction for the indicated time. Cells were collected, lysed and Aggregation Index was calculated for each time point as described in Figure 3. Data from 3 independent transformants, 3 triplicates each, are presented as mean ± SE.

One of the proteins that are firmly linked to aging is the NAD+-dependent protein deacetylase Sir2. Thus, we next followed the aggregation of polyQ proteins in a mutant lacking the *SIR2* gene. When compared to wild-type cells (Figure 3C), it is evident that despite similar expression level of the polyQ proteins, the aggregation of both 47Q and 103Q is increased remarkably in aging Δ*sir2* cells (Figure 5A, squares and triangles, respectively), and irrespective of *SIR2* deletion, 25Q still does not aggregate (Figure 5A, diamonds). Being a class III deacetylase that cleaves NAD^+^ for its activity, Sir2 releases nicotinamide (NAM), which is reported to be a noncompetitive inhibitor of Sir2 [69]. Indeed, in the presence of NAM, 47Q aggregation increases in aged cells (Figure 5B) in a concentration-dependent manner (Figure 5C). Even the highest concentration of NAM (40 mM) affects neither the already highly aggregated 103Q nor the never aggregated 25Q (Figure 5C).

Another aging-related protein, whose activity and content are critical for the cellular response to proteotoxic stresses, is Hsf1 [41]; [43]; [44]. When *HSF1* is overexpressed from a plasmid in wild-type cells, we find attenuated aging-dependent aggregation of the mid-size 47Q and the long 103Q, as compared to cells expressing an empty plasmid (pRS314) instead of p*HSF1* (Figure 5D). Our results indicate that Hsf1 is involved in aggregation of polyQ proteins in aging cells.

## Discussion

Neurodegeneration and aging, intimately interrelated processes, are the subject of intensive clinical research. The complexity of both processes, let alone when combined, makes it extremely difficult to elucidate the mechanisms that underlie aging-dependent neurodegeneration. Aging is poorly defined at the molecular level and the effects of aging on cellular processes are not fully understood. Nevertheless, many key genes implicated in longevity are highly conserved in evolution, suggesting that they operate in fundamental mechanisms. Neurodegenerative diseases are also diverse but one of their major hallmarks is the deposition of specific aggregation-prone proteins. To date neither the basis for the toxicity of these aggregates, nor their formation or the cellular processes affected by them is fully explained. Because of the close links between the two processes, models in mice, flies, worms and yeast were developed to study the effects of aging on protein aggregation and neurodegeneration.

In this study we expressed GFP-tagged proteins with different polyQ lengths in *S. cerevisiae* and followed their fate as a function of aging. We show that the short 25Q protein never aggregates and remains soluble at all times. The long 103Q protein is already aggregated very early after induction of its expression and levels of aggregates only slightly increase as the cells age. Our most interesting finding, shown in yeast for the first time, is that the mid-size 47Q protein is soluble and evenly dispersed in logarithmically growing yeast but begins to aggregate as the yeast cells enter the stationary phase. As the yeast cells aging progresses, the Aggregation Index (Figure 3) and the number of cells visualized with aggregates (Figure 4) increase further and sometimes even exceed the aggregation level of 103Q. Similar aging-dependent aggregation of mid-size polyQ proteins was also reported in *C. elegans* [19]; [68]. Our results demonstrate that the ability of yeast cells to maintain mid-size polyQ proteins in solution declines upon their aging. The sharp threshold in the onset of HD when huntingtin's polyQ tract contains around 45 residues (45Q) [22] suggests that our results with the near-threshold mid-size 47Q in aging yeast closely simulate the situation in humans. Thus, aging yeast may serve a reliable model system to study the dependence of aggregation on aging, a hallmark of many neurodegenerative disorders.

We show that yeast aging affects the aggregation of polyQ proteins and implicate aging-related genes in this phenomenon (Figures 3,4,5). Clearly, *HSF1* overexpression decreases aggregation of 47Q or 103Q (Figure 5D). These results may reflect an aging-dependent functional decline in Hsf1 and/or its many targets, mostly the chaperone network. Indeed, upregulation of chaperones increases longevity and enhances aging-related stress resistance in various models [70]. In particular, overexpression of Hsp70 extends the lifespan of *C. elegans* [71] and *Drosophila melanogaster* [72]. Likewise, a very recent screen of ∼900,000 small molecules has identified new classes of proteostasis regulators that induce HSF–1–dependent chaperone expression and restore protein folding in multiple conformational disease models [73]. It appears that proteostasis diseases are aggravated when the capacity of the chaperone network to cope with inherited misfolding-prone proteins, aging, or metabolic/environmental stresses declines [74].

Another major gene that has been implicated in aging in several organisms, including yeast where it was first discovered, is the class-III deacetylase Sir2, although its role in aging remains controversial. As noted above, there are two models of aging in yeast, RLS and CLS [75]. Whereas replicative aging may be a useful model for mitotically active cells, chronological aging represents more faithfully postmitotic cells, such as neurons. Interestingly, Sir2 is beneficial for RLS, whereas Δ*sir2* cells show higher CLS and better resistance to different stress conditions [40]. Here we find in aging Δ*sir2* cells increased aggregation (Figure 5A) that is also detected when Sir2 activity is blocked by NAM (Figure 5B, C).

Although further investigation is required to understand the role of Sir2 in aggregates formation in aging yeast, this phenomenon may reflect accelerated aging and failure to segregate damaged proteins in Sir2 mutants [76], possibly due to Sir2 involvement in maintaining the activity of the TRiC/CCT complex [77]. The ongoing debate of whether aggregates are protective or harmful to cells is still open. Uncoupling between aggregation and toxicity was recently reported from RNA interference genetic screens in *C. elegans* that have identified novel regulators of the proteostasis network [78]. Indeed, aggregates are observed in affected cells and serve as a hallmark of Alzheimer's, Parkinson's and HD. Also, in HD, polyQ aggregates are detected before the onset of clinical symptoms [79]. Yet, the formation of polyQ aggregates was reported to improve neurons survival compared to cells that had more diffuse distribution of huntingtin [80]. Moreover, analysis of polyQ structural changes showed that a soluble monomeric beta-sheet conformer of the expanded polyQ was a major cause of cytotoxicity [81]. Therefore, large aggregates may be the result of an active mechanism that has evolved to protect the cell by sequestering the toxic species, either the misfolded proteins and/or their oligomers [82]; [83]. It remains to be established how Sir2 and Hsf1 affect, directly or indirectly, the aggregation of polyQ proteins. Interestingly, deacetylation by Sirt1 (the closest mammalian homolog of Sir2) is reported to be one of the activation modes of the mammalian Hsf1 [51]. If yeast Hsf1 activity is also regulated by Sir2, this suggests a coordinated mechanism designed to handle aggregation-prone proteins.

The filter retardation aggregation assay we describe here (Figure 2) is simple, reproducible, and linear within a wide range of concentrations. Supported by the fluorescence microscopy (Figure 4), these assays allow monitoring the level of protein aggregation throughout the life of yeast populations in a quantitative fashion. Both assays can be easily applied to other cells and organisms. By these methods, and capitalizing on the ease and speed of genetic screening in yeast, this model system is particularly amenable to study the effects of genetic and environmental factors on aging and aging-dependent aggregation and determine their consequences on cytotoxicity and cell survival.

## Materials and Methods

### Yeast strains and plasmids

The yeast strains of *Saccharomyces cerevisiae* employed in this study are W303–1b (*MAT*α *ura3-52 trp1*Δ*2 leu2*–*3,112 his3*–*11 ade2*–*1 can1*–*100*) and KFY100 (*MAT*a *his4*–*619 leu2*–*3,112 ura3*–*52)*. Δ*sir2* (RS1717; W303–1b *sir2*Δ::*his5* ^+^) was generously provided by Prof. R. Sternglanz, Stony Brook University, USA [84]. The pRS314-*HSF1* plasmid was kindly provided by Prof. DJ Thiele, University of Michigan Medical School, USA.

The polyQ constructs used in this study were previously described [56]. They were generously provided by Prof. M. Sherman, Boston University, USA. The cDNAs encode the first 17 N-terminal amino acids of Huntingtin exon 1 (Htt_17_), followed by alternating CAG/CAA repeats of different lengths (encoding 25, 47 or 103 glutamine residues). These were fused in frame with a FLAG tag at the N-terminus, and green fluorescent protein (GFP) at the C-terminus, generating FLAG-Htt_17_-Q_25_-GFP (25Q), FLAG-Htt_17_-Q_47_-GFP (47Q) and FLAG-Htt_17_-Q_103_-GFP (103Q) (Figure 1A). The constructs were subcloned into the *URA3*-containging pYES2 vector, so the regulated expression of the polyQ proteins is under the galactose-inducible pGal1 promoter.

### Growth conditions

Yeast were regularly grown in minimal media which contained 0.67% (w/v) yeast nitrogen base, the appropriate nutrients required for selection of transformants, and 2% (w/v) glucose. For galactose induction, cells were transferred to medium containing 4% (w/v) galactose only, or 2% (w/v) galactose and 2% (w/v) fructose. Stationary phase cells that stopped dividing and reached 3.0–6.0 A_600_ (starting from 1.5×10^6^ cells/ml of glucose starters; cell number was calculated as 1A_600_ = 1.5×10^7^cells/ml) were grown for up to 15 days. All cells undergoing galactose induction were grown in 10 ml medium in 100 ml loosely-capped bottles, unless indicated otherwise.

For growth experiments, cells were grown logarithmically (0.6–1 A_600_) diluted to equal cell density (0.5 A_600_) and spotted as 10-fold serial dilutions on plates containing 2% (w/v) agar, 0.67% (w/v) yeast nitrogen base, the appropriate nutrients required for selection, and 2% (w/v) glucose or 4% (w/v) galactose. Alternatively, yeast cells were grown in glucose- or galactose-containing minimal liquid media, starting at 0.05 A_600_ and following their growth up to 0.8 A_600_, for calculating the duplication time.

### Quantitative fluorescence microscopy

Yeasts grown to logarithmic or stationary phase were viewed under the DMRBE fluorescence microscope (Leica). GFP was viewed using Chroma 41017 filter (excitation 470/40) and snapshots were taken using Magnafire 12-bit color CCD camera (Figure 4A). For aggregate quantification (Figure 4B), yeasts grown to logarithmic or stationary phase were imaged on a Nikon TiE fluorescent microscope with a 100X/1.49NA objective. GFP images of hundreds of cells were taken with 480/20 excitation filter, 525/40 emission filter (Chroma) using an Andor Clara 16-bit CCD camera. Images were processed using custom Matlab code [85]. Briefly, cells were segmented using the DIC channel. Fluorescence densities were computed by dividing total cell fluorescence by cell area. Independently, fluorescent foci were identified in the GFP channel using local adaptive thresholding, and then mapped to cells by location. Maximal density was computed from the fluorescence of the brightest focus in the cell divided by the focus area. The ratio *R* between the maximal density and the density is used as a measure for aggregation.

### Sample collection and alkaline lysis

Cell samples were collected at different times throughout the experiments, as indicated. Cell density was determined as A_600_, identical numbers of cells were collected by centrifugation (13,000 rpm, 1 minute, 4°C), washed with 0.01 M NaN_3_ in phosphate-buffered saline (PBS), and frozen (−20°C), to allow simultaneous lysis. Importantly, aggregates and protein levels were not affected by freezing (data not shown). Cells were lyzed by incubation for 30 minutes on ice in lysis buffer containing 0.2 M NaOH and 0.5% (v/v) β-mercaptoethanol, pH was adjusted to 8.0 with 5N HCl and samples were boiled for 5 minutes.

### Filter retardation and blotting assays

The protocol for measuring aggregation levels is based on modified versions of previously described methods: aggregates filtration [86] and total protein dot blotting. Equal amounts of boiled lysates were diluted 5-fold in either PBS or PBS supplemented with 2% (w/v) SDS (PBS/SDS). Samples in PBS were applied to PBS-soaked nitrocellulose membranes (0.2 μ; Protran Whatman) to absorb all proteins. Samples in PBS/SDS were filtered through nitrocellulose membrane soaked in PBS/SDS to retard only the aggregates. At least three independent transformants were analyzed and triplicate samples from each (total of 9 samples) were tested for absorbance and retardation assays. The data are presented as mean ± SE. All samples were applied to a 96-well dot blotter (Biorad). Membranes were blocked with 10% skim milk in PBS and polyQ proteins were detected by immunoblotting.

### Immunoblotting

The polyQ proteins were detected by a mouse anti-FLAG antibody (clone M2, Sigma). The secondary antibody used was IRDye 800CW-conjugated goat anti-mouse (LI-COR Biosciences). Alternatively, rabbit anti-GFP (ab290, Abcam) was used as a primary antibody followed by goat anti-rabbit IgG DyLight 680-labled (072-06-15-06, KPL). Secondary antibodies were visualized and quantified by the Odyssey Infrared Imaging System (LI-COR Biosciences).

## Supporting Information

Figure S1 **Quantitative microscopic assay reveals that 25Q never aggregates.** Wild-type cells (W303-1b) expressing 25Q were grown under galactose induction for up to 42 hrs. At each time point hundreds of cells were imaged by fluorescence microscopy. Images were analyzed for the presence of aggregates in individual cells as presented by the ratio between the maximal density (y axis) and the density (x axis).
(TIF)

Figure S2 **Quantitative microscopic assay reveals that aggregation of 47Q increases upon aging.** Wild-type cells (W303-1b) expressing 47Q were grown under galactose induction for up to 42 hrs. At each time point hundreds of cells were imaged by fluorescence microscopy. Images were analyzed for the presence of aggregates in individual cells as presented by the ratio between the maximal density (y axis) and the density (x axis).
(TIF)

Figure S3 **Quantitative microscopic assay reveals that 103Q is always aggregated.** Wild-type cells (W303-1b) expressing 103Q were grown under galactose induction for up to 42 hrs. At each time point hundreds of cells were imaged by fluorescence microscopy. Images were analyzed for the presence of aggregates in individual cells as presented by the ratio between the maximal density (y axis) and the density (x axis).
(TIF)
