# Supplementary figures and images for: Aggregation of PolyQ Proteins Is Increased upon Yeast Aging and Affected by Sir2 and Hsf1: Novel Quantitative Biochemical and Microscopic Assays
Source: PLoS One. 2012 Sep 6;7(9):e44785. doi: 10.1371/journal.pone.0044785 (PMC3435303; doi:10.1371/journal.pone.0044785)

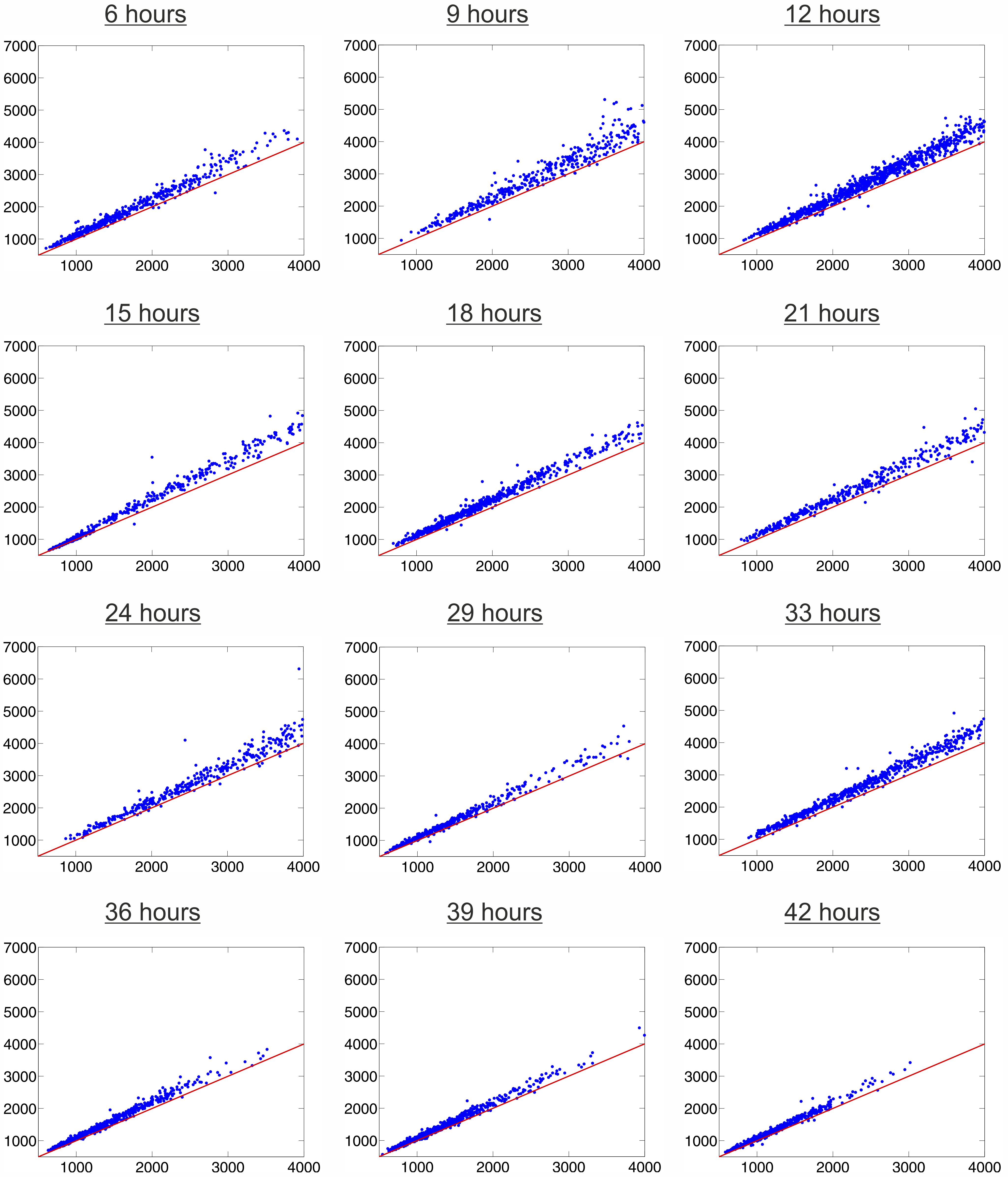

Supplement: Figure S1 — Quantitative microscopic assay reveals that 25Q never aggregates. Wild-type cells (W303-1b) expressing 25Q were grown under galactose induction for up to 42 hrs. At each time point hundreds of cells were imaged by fluorescence microscopy. Images were analyzed for the presence of aggregates in individual cells as presented by the ratio between the maximal density (y axis) and the density (x axis). (TIF) [file pone.0044785.s001.tif]

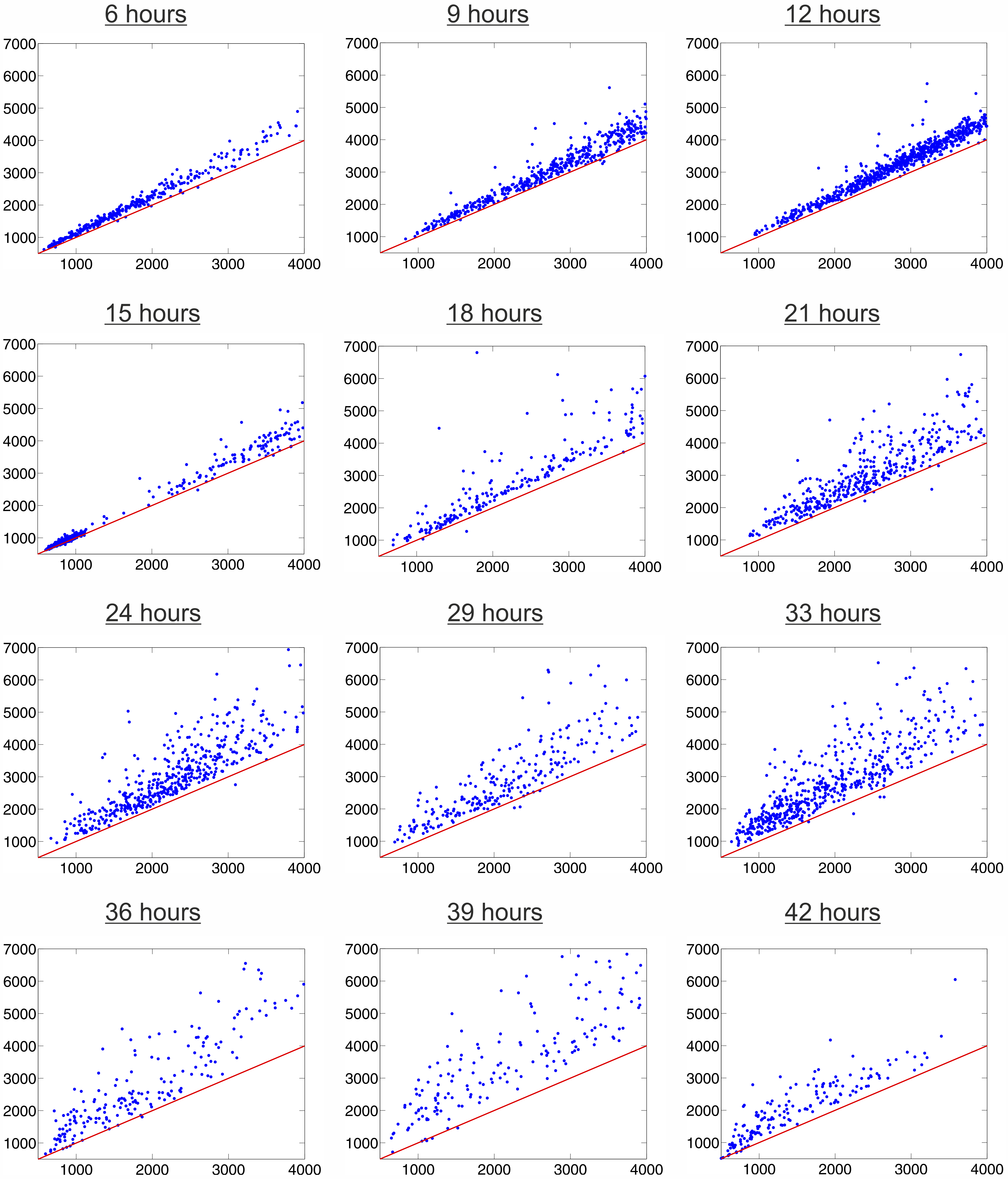

Supplement: Figure S2 — Quantitative microscopic assay reveals that aggregation of 47Q increases upon aging. Wild-type cells (W303-1b) expressing 47Q were grown under galactose induction for up to 42 hrs. At each time point hundreds of cells were imaged by fluorescence microscopy. Images were analyzed for the presence of aggregates in individual cells as presented by the ratio between the maximal density (y axis) and the density (x axis). (TIF) [file pone.0044785.s002.tif]

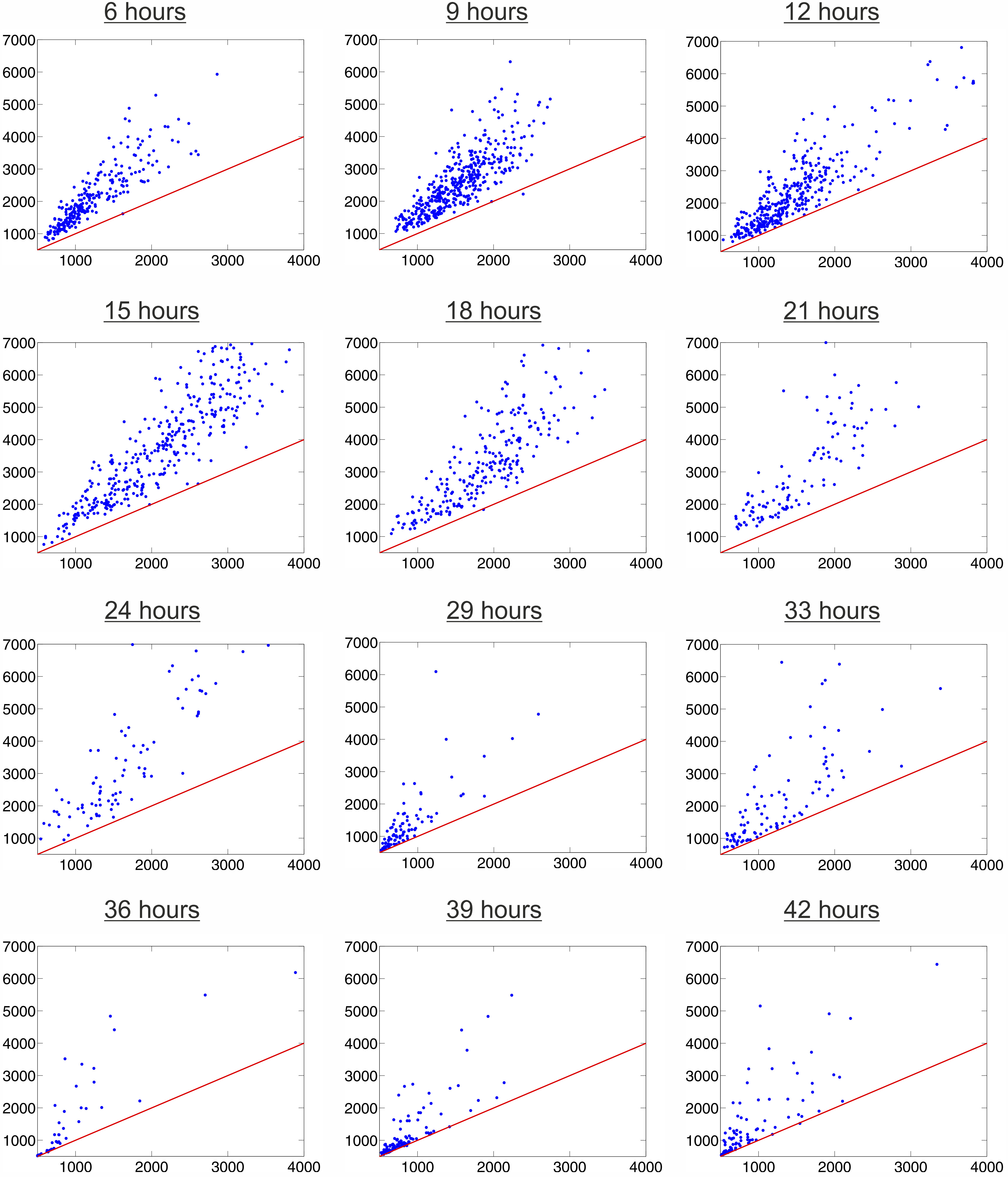

Supplement: Figure S3 — Quantitative microscopic assay reveals that 103Q is always aggregated. Wild-type cells (W303-1b) expressing 103Q were grown under galactose induction for up to 42 hrs. At each time point hundreds of cells were imaged by fluorescence microscopy. Images were analyzed for the presence of aggregates in individual cells as presented by the ratio between the maximal density (y axis) and the density (x axis). (TIF) [file pone.0044785.s003.tif]
